# [Retracted] Novel compound cedrelone inhibits hepatocellular carcinoma progression via PBLD and Ras/Rap1

**DOI:** 10.3892/etm.2025.13018

**Published:** 2025-11-17

**Authors:** Jiansong Wu, Qiang Niu, Jie Yuan, Xiaodan Xu, Liuxia Cao

Exp Ther Med 18:4209–4220, 2019; DOI: 10.3892/etm.2019.8080

Following the publication of this paper, it was drawn to the Editor’s attention by a concerned reader that the tumor images shown in Fig. 5A on p. 4218 were strikingly similar to data that had already appeared in different form in another article written by different authors at different research institutes, which was published in the journal *OncoTarget*.

In view of the fact that the abovementioned data had already apparently been published previously, the Editor of *Experimental and Therapeutic Medicine* has decided that this paper should be retracted from the Journal. The authors were asked for an explanation to account for these concerns, but the Editorial Office did not receive a reply. The Editor apologizes to the readership for any inconvenience caused.

